# Antimicrobial prophylaxis in decompensated cirrhosis: friend or foe?

**DOI:** 10.1097/HC9.0000000000000228

**Published:** 2023-08-31

**Authors:** Dominic Crocombe, Alastair O’Brien

**Affiliations:** Division of Medicine, UCL Institute for Liver and Digestive Health, London, UK

## BACKGROUND

Infection is a major driver of mortality and morbidity in decompensated cirrhosis, and the risk of both community-acquired and nosocomial infection correlates with increased severity of liver disease. Mechanisms underlying the increased risk of infection in cirrhosis include intestinal bacterial overgrowth, subsequent bacterial translocation from a relatively less mobile and more permeable digestive tract, and impaired immune responses due to hepatic synthetic dysfunction. Hepatologists appear to have a low threshold for prescribing antibiotics to patients with cirrhosis even in the absence of a firm diagnosis of active infection, with a secondary analysis from the multicenter ATTIRE (albumin to treat infection in patients with chronic liver failure) randomized controlled trial of patients hospitalized with decompensated liver disease demonstrating that 49.8% of patients who were prescribed antibiotics on admission did not have a diagnosis of infection.^1,2^

**FIGURE 1:**
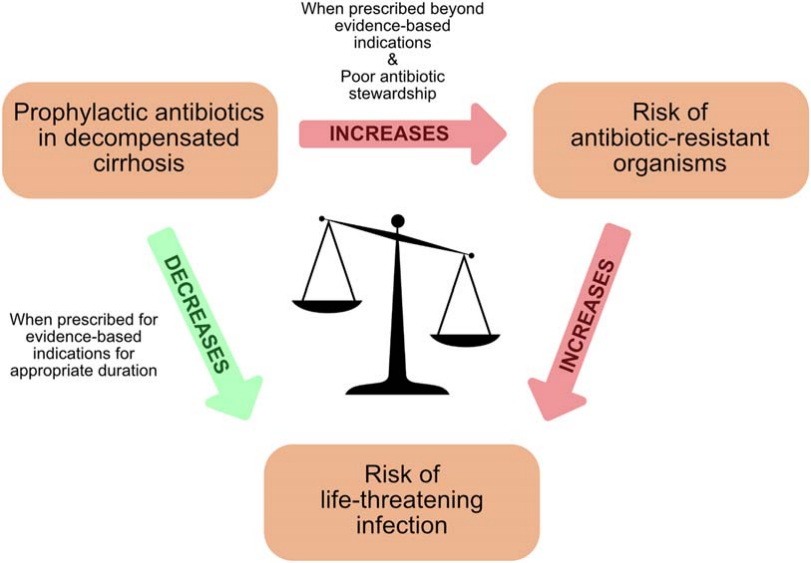
Antibiotic prophylaxis in decompensated cirrhosis, a balancing act.

Prophylactic antibiotics are often considered for those patients deemed most susceptible to developing infection and subsequent poor outcomes. However, all patients with decompensated cirrhosis are at a heightened risk and stratifying those at greatest risk is not necessarily straightforward. Furthermore, the rise of antibiotic use in medicine (and agriculture) is driving one of the major challenges facing health care, antimicrobial resistance (AMR).^3^ Hospitalization and antibiotic use are independent risk factors for the development of multidrug resistant (MDR) organisms.^4–6^ MDR organisms are now responsible for 25,000 deaths a year across the United States and the European Union overall.^7^ In cirrhosis, large studies have shown that MDR organisms represent over one third of organisms cultured in Europe and globally.^8,9^ This problem will only increase if the trend demonstrated in the Global Burden of Disease Study, which showed a significant increase in age-standardized prevalence of decompensated cirrhosis between 1990 and 2017,^10^ continues. With no new antibiotics on the immediate horizon (and this cannot be the solution, as new antibiotics are also likely to drive resistance), we have a duty to use these crucial drugs effectively and with care. Therefore, the potential benefits of antibiotic prophylaxis need to be balanced against the principles of good antimicrobial stewardship^11^ (Figure 1).

Antibiotics prescribed for prophylaxis should have demonstrated efficacy in high-quality clinical trials, have a low potential for AMR, be low cost and have few adverse effects. In our opinion, antibiotic prophylaxis can only be justified if there is a reasonable expectation that it will lead to improved length and quality of life for the patient. In this review, we assess the benefits and risks of antimicrobial prophylaxis in the clinical scenarios that characterize decompensated cirrhosis, and whether current evidence for prescribing antibiotics reaches this bar. We also identify areas for further research and, mindful of how challenging clinical trials in decompensated cirrhosis are to conduct, make suggestions as to how these might be undertaken.

## ASCITES

Antibiotic prophylaxis against spontaneous bacterial peritonitis (SBP) in patients with cirrhotic ascites is one of the widely accepted indications in standard hepatology practice. This is particularly true for patients who have already recovered from an episode of SBP, that is, secondary prophylaxis. Table 1 summarizes the clinical trials that have investigated the benefit of this practice on mortality, incidence of SBP, and other outcomes. It is very important to consider the 2018 Cochrane network meta-analysis of antibiotic prophylaxis to prevent SBP.^21^ This is a popular method that comes direct evidence from head-to-head comparisons of interventions from randomized controlled trials (RCTs) with indirect evidence from studies that compare different interventions with a common comparator.^22^ This is often used to compare interventions that have not been studied head-to-head. The authors compared the effects of 9 different antibiotic regimens versus no intervention from 29 different RCTs and reported there was “no evidence of differences between any of the antibiotics and no intervention in terms of proportion of people with ‘any adverse events’ (very low certainty), liver transplantation (very low certainty), or the proportion of people who developed SBP (very low certainty).”^21^ While SBP is generally regarded as the most common serious infection in patients with cirrhotic ascites,^23^ recent data from the ANSWER study showed that it occurred in 8% of participants whereas non-SBP bacterial infections occurred in 11.8%.^24^ This highlights the increased susceptibility to all infections faced by patients with decompensated cirrhosis. It also raises questions about whether the incidence of a specific infection (such as SBP) or broader clinical outcomes (such as all-cause mortality) are the most appropriate primary end points in clinical trials of antibiotic prophylaxis.

**Table 1:** Summary of clinical trials investigating long-term antibiotic prophylaxis versus no antibiotic for patients with cirrhotic ascites

Study	Main inclusion criteria	Intervention	n	Follow up	Mortality	SBP	Other outcomes	Effect on antibiotic resistance
Long-term prophylaxis to prevent SBP in patients with cirrhotic ascites								
Primary prophylaxis studies: antibiotic vs no antibiotic								
Novella, 1997	Low-protein ascites (≤10 g/L) or bilirubin >25 µg/L	Norfloxacin 400 mg/day vs Norfloxacin 400 mg/day in hospital only	56 53	11 months	23.3% NS 30.2%	1.8% p < 0.01 16.9%	No difference in rate of infections overall.	90% of E.coli isolated from infections in norfloxacin daily group, versus 36.3% in control group, were norfloxacin-resistant. Overall incidence of norfloxacin-resistant infections not significantly different (19.6% vs 15%).
Grangé, 1998	Low-protein ascites (<15 g/L)	Norfloxacin 400 mg/day vs Placebo	53 54	6 months	15.0% NS 18.5%	0.0% p < 0.05 9.4%	Severe infections overall: 1 (norfloxacin) vs 9 (placebo)	12 norfloxacin-resistant organisms isolated in norfloxacin group vs 3 organisms in placebo group.
Fernández, 2007	Low-protein ascites (<15 g/L) + advanced cirrhosis	Norfloxacin 400 mg/day vs Placebo	35 33	1 year NS	28.6% p < 0.05 39.3%	5.7% 30.3%	Norfloxacin: lower mortarlity at 3 months, lower incidence of HRS at 1 year.	84.6% (11/13) of Gram-negative bacilli infections (mainly urinary) in the norfloxacin group were quinolone-resistant vs 16.7% (1/6) in placebo group (p = 0.01).
Terg, 2008	Low-protein ascites (<15 g/L)	Ciprofloxacin 500 mg/day vs Placebo	50 50	1 year p < 0.04	12% NS 28%	4% 14%	Ciprofloxacin: lower incidence of bacterial infections overall (p = 0.05)	In 2 cases, ciprofloxacin-resistant E. coli was cultured in the ciprofloxacin group vs none in the placebo group.
Secondary prophylaxis studies: antibiotic vs no antibiotic								
Ginés, 1990	Previous SBP	Norfloxacin 400 mg/day vs Placebo	40 40	1 year	17.5% NS 25%	12% p = 0.01 35%	No difference in rate of infections overall.	No significant incidence of resistant organisms isolated
Mixed primary and secondary prophylaxis studies: antibiotic vs no antibiotic								
Soriano, 1991	Low-protein ascites (<15 g/L). <9% patients had prior SBP.	Norfloxacin 400 mg/day vs No intervention	32 31	Not reported	6.2% NS 16.1%	0 p < 0.05 22.5%	Overall infection rate 3.1% vs 41.9% (p<0.005). Incidencce of infection caused by Gram negative bacilli 0% vs 29%.	Not report
Singh, 1995	Cirrhotic ascites. <27% patients had prior SBP.	Trimethoprim-sulfamethoxazole 160/800 mg 5d/w vs No intervention	30 30	90 days	7.0% NS 20%	3.0% p = 0.025 27.0%	Overall infection rate 3% vs 30% (p = 0.01)	Not reported
Rolachon, 1995	Low-protein ascites (<15 g/L). <15% patients had prior SBP	Ciprofloxacin 750 mg/week vs Placebo	28 32	6 months	14.3% NS 18.8%	3.6% p < 0.05 22%	No difference in incidence of extraperitoneal infection.	No resistant organisms isolated in either group.
Moreau, 2018	Advanced cirrhosis (Childs C); <5% patients had prior SBP	Norfloxacin 400 mg/day vs Placebo	144 147	6 months NS	14.8% NS 19.7%	10% 17%	Norfloxaxcin: lower mortality only in patients with low-protein ascites (< 15 g/L). Fewer bacterial infections overall (p < 0.05) and fewer Gram negative bacteria cultured (p < 0.005).	No significant difference in the incidence of antibiotic-resistant bacterial infections between the groups (2 vs 1)

### Studies for secondary prophylaxis

Recurrent SBP confers a devastating risk of mortality,^25^ and secondary prophylaxis following an initial episode is widely recommended as part of standard care.^26–28^ The single trial that underpins this recommendation, a landmark placebo-controlled RCT in 80 patients reported by Ginés et al^16^ in 1990, demonstrated the effectiveness at 1 year of norfloxacin 400 mg/d at preventing SBP recurrence following an initial episode (risk reduction from 35% to 12%, *p* = 0.01). Specifically, it significantly reduced the risk of SBP caused by aerobic gram-negative bacilli, but not SBP caused by other organisms or culture negative SBP. No significant risk of developing MDR organisms in the treatment group was reported. Despite the clear reduction in SBP recurrence, it is important to note that there was no reduction in all-cause mortality, total incidence of infections, or other complications during the 1-year trial period. In the 33 years since publication of this trial, there have been no further RCTs solely studying secondary antibiotic prophylaxis versus placebo for patients following an episode SBP, such has been the scale of acceptance of secondary prophylaxis into standard clinical practice.

### Mixed studies

Three other RCTs from the 1990s studied antibiotic prophylaxis in mixed populations (ie, containing patients who had previously had SBP and patients who had not) and thus generated evidence regarding both primary and secondary antibiotic prophylaxis in parallel.^17–19^ The proportion of patients who had a prior history of SBP ranged from 9% to 27% in these 3 RCTs. These studies suggest that prophylaxis with antibiotics (norfloxacin 400 mg/d,^17^ ciprofloxacin 750 mg/wk,^19^, or trimethoprim-sulfamethoxazole (co-trimoxazole) 160/800 mg 5 days per week,^18^ respectively) reduces rates of SBP in patients with cirrhosis and ascites but has no effect on mortality. Two of the 3 mixed studies found a significantly lower rate of overall infections in the groups receiving antibiotics (<5% in the treatment groups compared with >30% in placebo groups) but neither of these reported the impact on MDR organisms.^17,18^ Also, these studies were small (maximum 32 patients per group in each study), and not without flaws: most notably, Soriano et al’s^17^ 1991 paper did not report the duration of follow-up.

More recently, Moreau et al^20^ reported the results of the NORFLOCIR trial, the largest placebo-controlled RCT investigating antibiotic prophylaxis in decompensated cirrhosis to date (n = 291). While this study also included a mixed population, <5% of patients had a prior history of SBP so the findings are arguably most relevant to the question of primary prophylaxis. The primary results were that norfloxacin 400 mg/d did not reduce overall mortality or incidence of SBP in this mixed population. However, results of a secondary analysis showed a significant mortality benefit in the subgroup of patients who had low ascitic fluid protein levels (<15 g/L).^20^ Nonetheless, despite the large scale of this study the intended sample size (n = 392) was not reached and so the outcomes should be interpreted with caution.

### Studies for primary prophylaxis

Primary antibiotic prophylaxis to prevent a first episode of SBP in patients with cirrhotic ascites is a less well-established practice. As 90% of SBP episodes occur in patients with no prior SBP,^29^ the question of whether this approach is beneficial represents a major gap in the evidence. Many clinicians use the ascitic protein level to stratify the risk of developing SBP because low ascitic protein concentrations (≤15 g/L) are associated with a greater risk of SBP, potentially due to reduced host opsonization activity against bacterial pathogens.^30^ This approach was reinforced in the aforementioned NORFLOCIR trial.^20^ However, it is possible that low ascitic protein concentration simply reflects disease severity or other confounders and the validity of low-protein ascites as a reliable risk factor for SBP has been challenged recently by 2 large post hoc analyses (of 591 and 683 patients, respectively) that showed no increased risk of SBP in those with low-protein ascites.^31,32^

Guideline-producing bodies are also split on this topic. Both the National Institute for Health and Care Excellence^26^ and the European Association for the Study of the Liver (EASL)^28^ recommend primary prophylaxis only for patients whose ascitic protein concentration is <15 g/L. The American Association for the Study of Liver Diseases recommend it only if ascitic protein is <15 g/L *and* the patient has advanced liver failure (defined by Child-Pugh score >9 and bilirubin ≥3 mg/dL) or renal dysfunction (serum creatinine >1.2 mg/dL, urea >25 mg/dL, or sodium <130 mEq/L).^27^ The British Society of Gastroenterology does not provide guidance on primary prophylaxis due to a lack of consensus.^33^

### The ASEPTIC trial

The ASEPTIC trial (Primary Antibiotic prophylaxis using co-trimoxazole to prevent SpontanEous bacterial PeritoniTIs in Cirrhosis) is a multicenter RCT designed to address this question, with results anticipated in 2025.^34^ Fluoroquinolones were not selected following the MHRA warning (see later) and it was considered that a trial of rifaximin would have faced major operational challenges as this is used widely for HE and it is considerably more expensive. Data on all infections are collected but the primary endpoint is overall survival and having recruited >390 patients from 41 sites to date there will be sufficient power to detect a clinically significant difference. Quality of life data is also being collected. Twenty-five percent of recruited patients to date were actively drinking alcohol and this is one of the stratification variables. Furthermore, the analysis will include an assessment of alcohol cessation/recidivism during the trial as this is likely to be the most important confounder, as alcohol accounts for the vast majority of the causes of cirrhosis. Originally, ascitic protein <2 g/dL was proposed as an inclusion criterion for this trial (as many hospital laboratories only reported this threshold), however, in the pilot phase we found a low number of cases of low ascitic protein (38 out of 224 patients), despite only screening patients with refractory ascites and advanced liver disease. We considered that restricting primary prophylaxis to those with Child-Pugh ≥9, serum bilirubin level ≥3 mg/dL, with either impaired renal function or hyponatremia was too restrictive as many of these patients would be very unlikely to recover without transplantation. We therefore based our inclusion criteria on the ANSWER study^24^ and have included patients with Child-Pugh class B or C, and the presence of ascites requiring any diuretic treatment or at least 1 or more paracentesis within 3 months before enrollment. We believe that these inclusion criteria will recruit patients with significant mortlaity (34% at 18 months) but who do have the potential to recover, rather than patients that have reached the point for end of life care, for whom an interventional trial would not be ethical. Subgroup analysis will be performed to examine outcomes in patients with an ascitic protein content ≥2 and <2 g/dL. Trial medication is stopped if the patient re-compensates their ascites or reaches end-of-life care. The trial treatment period of 18 months will test whether any benefit wanes with time, as has been proposed.^35^ Finally, we are collecting data on AMR.

### Choice of antibiotic

A range of antibiotic regimens have been studied but there is no clear consensus about superiority. In the 2018 Cochrane review, with reference to “any adverse events” occurring in the 29 RCTs included, there was a trend toward better outcomes for co-trimoxazole (rate ratio=0.19) and norfloxacin (rate ratio=0.74) over other antibiotics but these data were from small trials of low certainty.^21^ Several other RCTs have compared the efficacy of different antibiotics for this purpose head-to-head, with few showing significant superiority of one antibiotic class over another (Table 2). In clinical practice, antibiotic prophylaxis must be guided by the patient’s specimen culture history, local sensitivities, and patient factors such as allergy profile, and balanced against the risk of driving AMR. Patients should be informed of the potential risks and benefits, the importance to take as prescribed, and to stop if prescribed another antibiotic for a different indication.

**Table 2:** Summary of clinical trials comparing different antibiotics for long-term prophylaxis in patients with cirrhotic ascites

Study	Main inclusion criteria	Intervention	n	Follow up	Mortality	SBP	Other outcomes	Effect on antibiotic resistance
Long-term prophylaxis to prevent SBP in patients with cirrhotic ascites								
Studies comparing antibiotic vs antibiotic								
Bauer, 2002	Previous SBP	Rufloxacin 400 mg 3 days per week for 1 week then weekly vs Norfloxacin 400 mg/day	39 40	1 year	12.8% NS 7.5%	31% p = 0.09 15%	No difference in incidence of extraperitoneal infection.	In rufloxacin group 2/7 organisms isolated were quinolone resistant
Alvarez, 2005	Previous SBP, low-protein ascites (≤10 g/L), and/or bilirubin >25 µg/L	Nofloxacin 400 mg/day vs Trimethoprim/sulfamethoxazole 160/800 mg 5 day/week	18 17	6 months	21.90% NS 20%	9.40% NS 16%	No difference in incidence of extraperitoneal infection.	1 case of norfloxacin-resistant organism causing infection in the norfloxacin group (none in the other).
Pande, 2012	Previous SBP, low-protein ascites (<10 g/L), and/or bilirubin >25 µg/L	Norfloxacin 400 mg/day + probiotics vs Norfloxacin 400 mg/day + placebo	55 55	6 months	23.6% NS 25.4%	34.5% NS 36%		Not reported
Lontos, 2014	Previous SBP, low-protein ascites (<15 g/L), and/or bilirubin >25 µg/L	Norfloxacin 400 mg/day vs Trimethoprim/sulfamethoxazole 160/800 mg/day	40 40	1 year NS	5.0% NS 5.0%	27.5% 17.5%	No difference in infection rate overall.	5 out of the 10 cultures isolated demonstrated antibiotic resistance to the antibiotic given
Assem, 2016	Low protein ascites (<15 g/L) with advanced liver disease (Child-Pugh ≥9 and bili ≥30) or renal impairment (creatinine ≥1.2 mg/dL, urea ≥25 mg/dL, or sodium <130)	Norfloxacin 400 mg/day vs Rifaximin 110 mg/day vs Rifaximin 110 mg/day, then Norfloxacin 400 mg/day, for alternating months	78 82 79	6 months	13.9% NS 9.8% NS 7.6%	22.8% NS 12.5% NS 9.2% (p=0.04 vs norflox alone)	No differences between groups regarding adverse events or other outcomes	not reported
Elfert, 2016	Previous SBP	Rifaximin 1200 mg/day vs Norfloxacin 400 mg/day	131 131	48 weeks	13.7% p=0.04 24.4%	3.9% p = 0.04 14.1%	Significantly fewer side effects reported with rifaxmin.	8/8 positive cultures of ascitic fluid in patients with new SBP were resistant to norfloxacin
Yim, 2018	Previous SBP, or low-protein ascites (<15 g/L) Primary prophylaxis sub-group: Low-protein ascites (<15 g/L)	Norfloxacin 400 mg/day vs Ciprofloxacin 750 mg/week Norfloxacin 400 mg/day vs Ciprofloxacin 750 mg/week	62 62 56 52	12 months NS NS	27.3% NS 26.3% 24.0% NS 27.1%	7.3% 5.3% 2.0% 4.2%	No differences between groups	A pathogenic organism was isolated in only 1 patient (in the ciprofloxacin arm) and this was a quinolone-resistant E. coli.
Praharaj, 2022	Primary prophylaxis sub-group: Low-protein ascites (<15 g/L) and advanced liver failure or renal dysfunction Secondary prophylaxis sub-group: Previous SBP	Rifaximin 550 mg twice/day vs Norfloxacin 400 mg/day Rifaximin 550 mg twice/day vs Norfloxacin 400 mg/day	33 33 33 33	6 months NS NS	24.2% NS 18.2% 21.2% p < 0.01 36.4%	14.3% 24.3% 7.0% 39.0%	No differences between groups Lower incidence of HE in the rixafimin group (23.1% vs 51.5%, p = 0.02)	Not reported Not reported

The most widely recommended and studied class of antibiotic for prophylaxis against SBP are quinolones. However, the antibiotic with the largest evidence-base, norfloxacin, is not available in the United Kingdom or the United States. Growing concern regarding adverse events of other fluoroquinolones has led to restrictions and warnings being placed by the US Food and Drug Administration, European Medicines Agency, and MHRA.^44^ These were based on reports of adverse reactions, such as tendonitis or tendon rupture, muscle pain, muscle weakness, joint pain, joint swelling, peripheral neuropathy, and central nervous system effects and increased risk of aortic aneurysm rupture.

Furthermore, a clear relationship has been demonstrated between excessive quinolone use and the steady increase in the incidence of quinolone-resistant bacterial pathogens, both in hospital and community sites.^45^ Their excessive use has coincided with increases in the prevalence of quinolone resistance among nosocomial gram-negative bacilli as well as gram-positive cocci linked with community-acquired infections.^46^ In addition, excess exposure to quinolones has been associated with colonization and infection by health care–associated pathogens such as methicillin-resistant *Staphylococcus aureus* and *Clostridium difficile*.^47^ Moreover, quinolone usage may contribute significantly to the emergence of resistance to other classes of antibiotics, such as carbapenems.^48^

US guidance recommends either norfloxacin or co-trimoxazole for primary SBP prophylaxis in those with severe liver failure and/or renal dysfunction.^27^ Co-trimoxazole is less studied but has been shown to have similar efficacy to norfloxacin in preventing SBP. It may also be less likely to drive AMR as it has been widely used in resource-poor settings for HIV patients with little evidence of emergent resistance to it.^49^ There is also evidence of its cost-effectiveness in this setting.^50^ UK data comparing co-trimoxazole to norfloxacin for primary prophylaxis which showed similar efficacy but no admissions for *C. difficile* diarrhea in the co-trimoxazole group compared with 13/134 in the norfloxacin group.^51^ Potential adverse events associated with co-trimoxazole are hyperkalemia, blood cell dyscrasias, and the very rare Stevens-Johnson syndrome, and caution is recommended in renal dysfunction.

Rifaximin, as a nonabsorbable antibiotic, has low rates of resistance and may have potential utility for SBP prophylaxis, however, not all studies have shown benefit^52–54^ and it is considerably more expensive than the alternatives. Amoxicillin/clavulanic acid is an alternative and has been used for alcohol-associated hepatitis, but it has not been widely studied in this context and there are concerns over DILI. Some studies employed intermittent or alternating courses of antibiotics. However, on balance, there is little convincing evidence that such approaches are superior in terms of efficacy, safety, or adherence.

### When to stop

Evidence-based strategies of how to minimize the risk of AMR are also lacking. For example, the EASL guidelines state norfloxacin prophylaxis should be stopped in patients in whom the ascites disappears, but it is left to clinical judgement to determine duration of therapy in patients with persistent ascites.^28^ We believe a logical approach would be to stop antibiotics if the ascites resolves or a patient undergoes TIPS or, of course, liver transplantation. Clinicians might consider switching antibiotic if the patient is hospitalized with an infection resistant to the prophylaxis treatment. Antibiotics should be stopped if the patient deteriorates and is deemed to be in the end phase of their life; we believe this includes insertion of indwelling ascitic drains for palliative care and recommend stopping prophylactic antibiotics for these patients (Table 3).

**TABLE 3:**
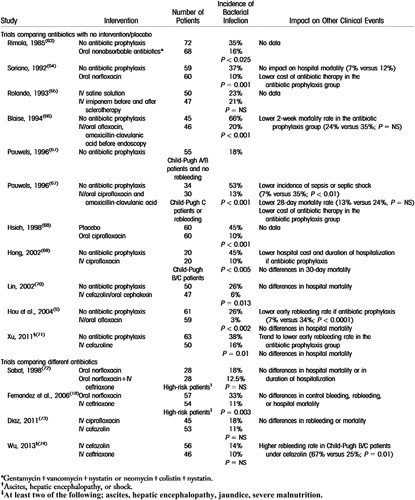
Summary of clinical trials investigating antibiotic prophylaxis versus no antibiotic for patients with acute gastrointestinal variceal bleeding

Abbreviation: NS, not significant.

Table taken directly from Fernández et al.^55^

## ACUTE GASTROINTESTINAL VARICEAL BLEEDING

Prompt antibiotic prophylaxis has been an established part of standard care in acute variceal bleeding for several decades, conferring short-term benefits against mortality, rebleeding, and secondary infection.^56,57^ The clinical trials investigating antibiotic prophylaxis in this scenario were summarized by Fernandez et al in 2016^55^ (Table 3) and we are not aware of any further RCTs that have been conducted since. Although many of the trials used third-generation cephalosporins, no specific antibiotic is recommended in most clinical guidelines. While bacterial infections can precipitate acute variceal bleeding, there is currently no role for prophylactic antibiotics in patients with cirrhosis and varices beyond the context of acute bleeding.

The rationale for antibiotic prophylaxis in acute bleeding has been proposed as reducing gut bacterial translocation, however, a recent multicenter cohort of patients hospitalized with acute variceal bleeding found the most common infections were lower respiratory tract, for which reducing bacterial translocation would appear to be far less important than SBP. Indeed, this study also found that 19.3% (320/1656) developed bacterial infection despite antibiotic prophylaxis.^58^ Furthermore, the most recent trial included in the meta-analyses that form the basis of international guidelines are from 2006^59^ and endoscopic therapy has improved substantially since the studies included.^60^ It may be that antibiotic prophylaxis could be prescribed only to those with the most severe liver disease, but in view of the current guidelines recommending prophylaxis for all patients, a clinical trial to investigate this approach would encounter ethical challenges.

We suggest a short duration of antibiotics since there is no rationale in these circumstances to “complete the course.” In patients with Child-Pugh score <7 that have had successful endoscopic therapy antibiotics could be stopped immediately. Indeed, we suggest many of these patients do not need to start antibiotics and would reserve them for patients with large volume blood loss. For those with Child-Pugh >7, antibiotic prophylaxis should be stopped at 48–72 hours if there is no rebleeding or need for rescue therapy (eg, TIPS), and no sign of infection/sepsis or organ failure.

## OTHER (POTENTIAL) INDICATIONS

### Hepatic Encephalopathy

Previously, the use of nonabsorbable antibiotics in the treatment of active HE was based on small clinical trials (n = 33) conducted in the 1970s.^61^ A large placebo-controlled RCT (n = 299) in 2010 demonstrated that rifaximin was effective at reducing recurrence of HE over a 6-month period (HR=0.42) in patients who had previously had HE, that is,for secondary prophylaxis.^62^ In this context, antibiotic therapy is not preventing infection per se but minimizing the growth of ammonia-producing bacterial species in the gut flora. The recently updated EASL guidelines on the management of HE recommend rifaximin (in addition to lactulose) as secondary prophylaxis following 2 or more episodes of overt HE within a 6-month period.^63^ This is based on the aforementioned RCT plus a meta-analysis that demonstrated its effectiveness in this context (relative risk=1.32).^64^ EASL also recommend consideration of rifaximin as secondary prophylaxis in patients who have had episodes of overt HE and are being considered for TIPS for a nonurgent indication. This is based on a placebo-controlled RCT of 197 patients that demonstrated a reduced risk of developing overt HE in the 168 days post-TIPS (OR=0.48) in the group who received rifaximin before the procedure.^65^ Whether antibiotic prophylaxis is beneficial for preventing first episodes of HE in patients with cirrhosis following other decompensation events such as acute variceal bleeding is unknown and unstudied.

### Acute-on-chronic liver failure

Infections are one of the most common precipitators of acute-on-chronic liver failure (ACLF)^66^ and so the antimicrobial prophylaxis approaches already discussed in this review may be considered strategies to prevent ACLF and specific antimicrobial prophylaxis strategies have been proposed in patients with ACLF.^9^ A recent trial of primary norfloxacin prophylaxis effectively prevents bacterial infections in patients with ACLF that recruited 172 patients in India.^67^ Mortality also significantly improved in the treatment arm,^68^ but more patients developed candiduria in the norfloxacin group. In contrast, the AntibioCor study of 284 patients with severe alcohol-associated hepatitis showed that the addition of amoxicillin/clavulanate to prednisolone reduced infection rates but did not extend survival and the authors do not recommend prophylaxis for these patients.^69^

### Awaiting liver transplantation

The only therapy proven to prolong life in patients with decompensated cirrhosis is liver transplantation, but due to the scarcity of the resource, this only occurs in a minority of cases.^1,70^ Patients on transplant waiting lists perhaps represent the cohort in whom considering antimicrobial prophylaxis for indications beyond the existing evidence-base is most enticing. After all, it is arguably to them that acute infections pose the greatest threat since any acute illness could threaten their eligibility for surgery. In contrast, these patients are also especially vulnerable to the threats posed by antimicrobial-resistant organisms. Colonization with these is associated with increased mortality for patients on transplant waiting lists.^71^ Therefore, we do not recommend prophylaxis in these patients beyond current guidance on primary/secondary prophylaxis for SBP or treatment of acute variceal hemorrhage.

## IDENTIFYING WHO IS LIKELY TO BENEFIT

Current guidelines recommend consideration of antibiotic prophylaxis in those patients likely to be at greatest risk of developing bacterial infection, namely those who have recovered from an episode of SBP, those experiencing variceal hemorrhage or those with low-protein ascites.^26–28,33^ There is rarely a specific duration of therapy recommended and patients often remain on antibiotics for a long time. Furthermore, there is little written about other characteristics or criteria that may be used to identify which patients are most likely to benefit.

Given the serious risk of AMR, we believe recommendations should be more specific. For example, if we consider a patient with decompensated alcohol-associated cirrhosis and ascites who has chosen to continue drinking alcohol, the potential clinical benefit of long-term antibiotic prophylaxis for this individual is unlikely to outweigh the negative impact of continued alcohol intake combined with the risk of developing MDR organisms. Equally, those patients who are extremely old and frail, or with a short life expectancy for other reasons, for example, co-existent malignancy, may have little to gain. Certainly, it seems counterintuitive to prescribe antibiotics in the absence of infection for patients receiving end-of-life care or for those with a long-term ascitic drain in situ for palliation.

In contrast, patients listed for liver transplantation or transjugular intrahepatic portosystemic shunt insertion may gain significantly from antibiotic prophylaxis. In such cases the indication is clear: to maximize disease-free time as a bridge to life-prolonging intervention and importantly prophylaxis is for a finite period. Likewise, patients with advanced alcohol-associated cirrhosis who successfully stop drinking alcohol are far more likely to benefit from prophylactic strategies than patients who continue to drink.

Therefore, until trial data are available to suggest otherwise, we recommend prophylaxis (for SBP and acute variceal bleed) is considered for the following patients with Child-Pugh >7 and presence of clinically significant ascites:

- Patients on the liver transplant or transjugular intrahepatic portosystemic shunt waiting list.
- Patients committed to alcohol cessation.
- Patients receiving treatment that may improve their underlying disease (eg, HCV, autoimmune hepatitis).
- Patients with potential for re-compensation or who might be considered for transplant or transjugular intrahepatic portosystemic shunt in the medium term.

We recommend prophylaxis should not be recommended for the following patients as the risk of AMR is high and there is unlikely to be any clinical benefit:

- Patients receiving end-of-life care.
- Patients with indwelling long-term ascitic drain for palliative care.
- Patients unable to attempt alcohol cessation.
- Patients with limited life expectancy (≤1 y).

## SUGGESTED APPROACH IN ACUTE DECOMPENSATED CIRRHOSIS

In the acute setting, clinicians often prescribe empirical antibiotics to hospitalized patients with decompensated liver disease even in the absence of likely infection or clear indication for prophylaxis (eg, acute gastrointestinal variceal bleeding). Data from the ATTIRE trial demonstrated that approximately half of all patients prescribed antibiotics on admission to hospital had no sign of infection.^2^ As this was a national RCT of 777 patients from 35 hospital sites across the United Kingdom, the prescribing practices are likely to be represent typical UK practice. Similarly, the organisms cultured from patients with confirmed infections are of interest. The commonest antibiotics prescribed in patients without a clinical diagnosis of infection were piperacillin-tazobactam (81/242, 33.4%) and amoxicillin/clavulanic acid (61/242, 25.3%) (Table 4). The spectrum of both these antibiotics covered most of the organisms cultured from hospital-acquired infections from the same trial cohort (Table 5).

**TABLE 4:** The antibiotics prescribed at ATTIRE trial baseline in patients without a clinical diagnosis of infection

Type of antibiotic prescribed	Number of prescriptions
Piperacillin/tazobactam	81
Co-amoxiclav (amoxicillin/clavulanic acid)	61
Ciprofloxacin	23
Ceftriaxone	18
Metronidazole	16
Amoxycillin	11
Cefotaxime	9
Co-trimoxazole	5
Levofloxacin	5

Two prescriptions each for vancomycin, clarithromycin, cefuroxime, teicoplanin, and trimethoprim.

One prescription each for flucloxacillin, temocillin, and gentamicin.

From Supplementary Material of Kutmutia et al.^2^

**TABLE 5:** Microbial organisms cultured from hospital-acquired infections in patients enrolled in the ATTIRE trial

Type of organism	Number of positive cultures
*Escherichia coli*	7
*Enterococcus*	7
*Staphylococcus*	7
Coliform	4
Gram-positive cocci	3
*Klebsiella*	2
*Streptococcus*	2

One each for yeast, *Clostridium difficile*, *Aspergillus*, gram-negative *Bacillus*, *Acinetobacter*, *Enterobacter cloacae*, and *Pseudomonas*.

From Supplementary Material of Kutmutia et al.^2^

**FIGURE 2:**
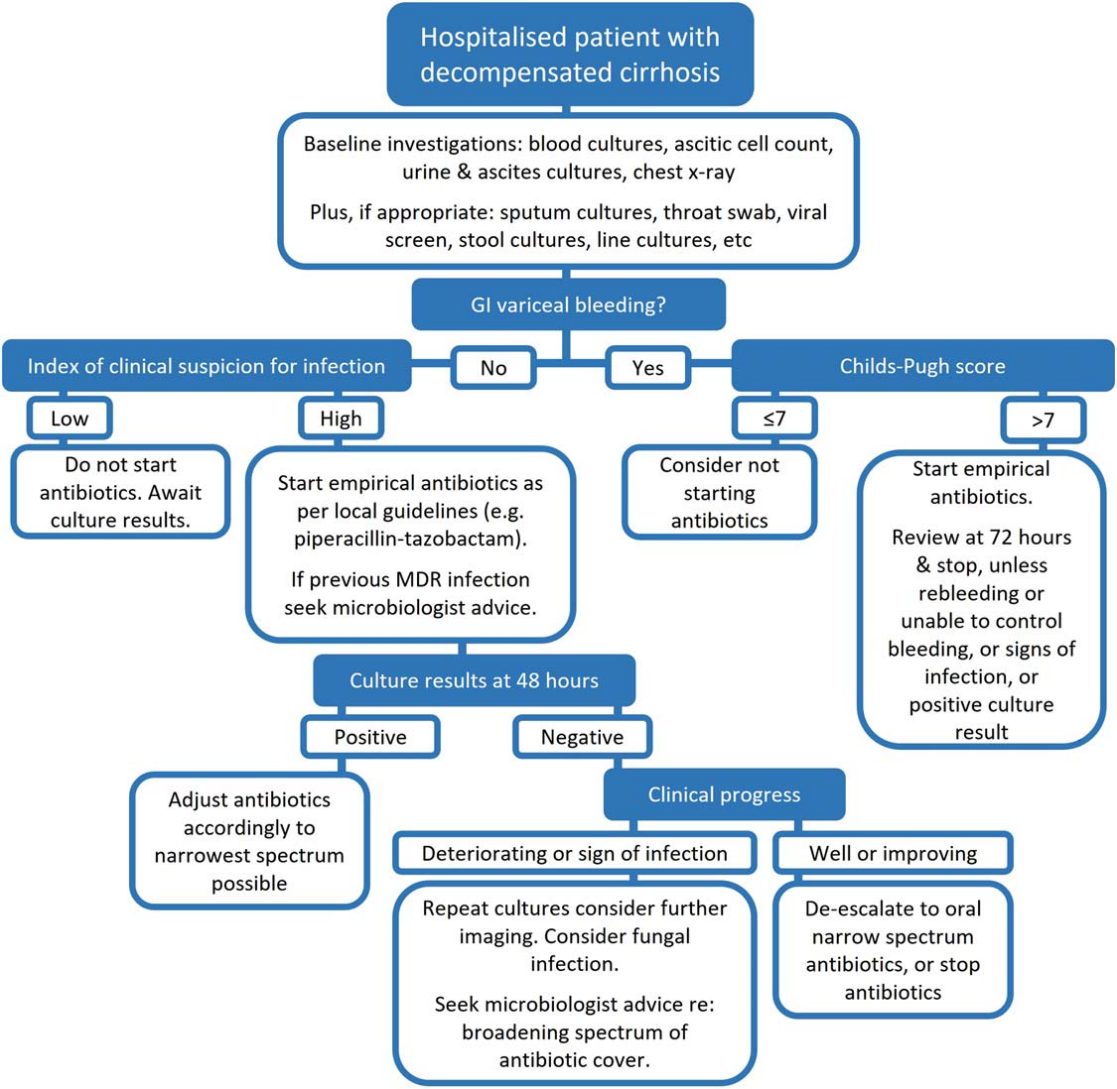
Suggested approach to empirical antibiotic therapy in hospitalized patients with decompensated cirrhosis. Abbreviations: GI, gastrointestinal; MDR, multidrug resistant.

With all considered, we suggest here a pragmatic and practical approach to antibiotic prescribing in the acute setting for hospitalized patients with decompensated cirrhosis (Figure 2). For a UK cohort, commencing empirical treatment with piperacllin/tazobactam and narrowing this to amoxicillin/clavulanic acid or stopping after 48 hours would seem a sensible approach if the patient is improving and no positive cultures are grown. Other antibiotics will be more appropriate in countries with high MDR rates. Liaising with local microbiology teams is strongly encouraged, especially when patients have a history of positive specimen cultures and/or their clinical response to treatment is poor.

## FUTURE PERSPECTIVES AND RESEARCH AVENUES

We must conclude that antimicrobial prophylaxis is more foe than friend unless accompanied by good clinical evidence of net benefit and, unless adequately addressed, we could unwittingly find ourselves in a postantimicrobial era.

Advances in data collection and monitoring systems might enable liver-specific microbial surveillance systems that could offer tailored antibiotic guidance in the future. This is frequently recommended in guidelines but existing data are at national or regional levels and cross-speciality.

Appropriately powered clinical trials are the best method to provide evidence; however, these are extremely challenging in patients with advanced liver disease. An early cessation of antibiotics trial would require a nonefficacy design, which would likely require a very large numbers of participants. Studies must include outcome measures of AMR to ensure any novel antimicrobial prophylactic strategies are safe and effective. It is hoped that data-enabled trials using routinely collected data such as hospitalization and mortality may make recruitment and retention more straightforward in the future. Equally, if overall survival is selected as the primary endpoint, then it could be argued that blinding would not be required, which would reduce placebo-related costs as overencapsulation is expensive, and the tablets are larger making it difficult for the patient to swallow. A factorial design could be used to improve efficiency that might enable a combination of systemically absorbed antibiotics and rifaximin to be tested in the same trial. Alternatively, a multiarm adaptive trial design to test multiple strategies, in which the high end of expectation of outcome is set as the threshold for stopping, might be effective but would require large trial networks. Increasingly, efficient trial designs are anticipated to improve the hepatology community’s ability to provide evidence to improve clinical practice.

## CONCLUSIONS

Currently, we overprescribe antibiotics based on evidence that in the current era should be considered outdated and derived from poor quality, underpowered trials, with a few exceptions. That is not to criticize these studies, but most belong to a previous era and as doctors we must continually reflect, update, and improve. Many of us adopt an approach centered around the belief that prescribing antibiotics to an individual with decompensated cirrhosis is a good thing and accept that we overprescribe but nobly suggest this is in our patients’ best interests and indeed many patients have come to expect this. However, antibiotics are far too important to abuse in this way and we will not be forgiven by future generations if they cannot rely on these wonder drugs because of our actions. Antibiotics should be reserved for treatment of a culture positive infection, unless there is high-quality evidence that proves otherwise. We have provided a pragmatic framework for current practice which focuses on stopping antibiotics early and we eagerly await new developments in this important area for clinical practice.

Antimicrobial prophylaxis is currently recommended by international guidelines for patients with decompensated cirrhosis in specific clinical scenarios, namely, to prevent infection in acute variceal bleeding, as secondary prophylaxis following an episode of SBP, as primary prophylaxis in only a small subgroup of patients with ascites, and as secondary prophylaxis against recurrent HE. Because infection is a notorious driver of morbidity and mortality in decompensated cirrhosis, clinicians may find it tempting to consider antimicrobial prophylaxis for other indications. However, our current prescribing practices surely represent a significant contributor to the global crisis of AMR and extending the use of these precious drugs when unsupported by sound evidence is likely to do more harm than good. Furthermore, it may be unwise to consider long-term antibiotic prophylaxis in patients with alcohol use disorder that have been unable to cut down their consumption, and certainly prophylaxis should not be prescribed in people receiving end-of-life care. However, many clinicians find such rationing of medication challenging and we must work together to ensure our practice is based on evidence. Adherence to the principles of good antibiotic stewardship is essential in clinical practice.

## CONFLICTS OF INTEREST

The authors have no conflicts to report.
